# Deception by Medical Assistance

**Published:** 1915-05

**Authors:** 


**A Monthly Review of Medicine and Surgery**

EDITOR AND PUBLISHER, DR. A. L. BENEDICT, 228 Summer St., corner of Elmwood Ave., Buffalo, (Address for all communications. Please make personal and telephone calls before 1 P. M.)

Third, in age, on the western continent. Independent, but supports professional organization. News limited mainly to Western N. Y. and Penn.

Subscription $2.00 a year; $3.00 for two years in advance. Changes and errors in address or failure or duplication of delivery, should be reported immediately.

Yearly Volume 70 MAY, 1915 No. 10

### Deception By Medical Assistance.

We adhere strongly to the theory of privileged communications and believe that, excepting instances in which the physician is required under legal compulsion to disclose professional secrets—possibly not even then unless actual harm will be produced by silence—it is not his duty to furnish testimony however he may be inclined to assist the operation of the law or to protect an injured party. For example, many cases of suicide are popularly believed to be hidden under death certificates supplied by accommodating physicians. So long as it is merely a matter of avoiding notoriety little practical harm is done. If the death certificate is falsified to secure insurance, whatever our tendencies to sympathise with the individual against a corporation, we can not get around the fact that the physician has perjured himself to enable one party to defraud another. Notification of communicable diseases, in general, involves no disgrace, no danger, often no expense. It is a procedure which in the long run, has protected and will protect, the very ones who seek to evade quarantine so that there can be no question but that the theory of privileged communication should yield to the legal requirement. In regard to venereal diseases, secrecy is more important from the standpoint of the individual afflicted, as well as of his family, although secrecy may actually endanger his family—especially a wife. Provided the person afflicted is conscientious with regard to the danger of infecting others, and provided that there is no law requiring the reporting of these diseases, there is good reason to respect the theory. But, how shall the physician act when the law explicitly requires such reporting, or, a fortiori when the patient is reckless as to spreading infection, perhaps actually endangering those to whom the same physician owes an equal duty as medical advisor?

Regarding abortions, alluding only to those in which a reputable physician has been called to attend a case in which abortion has already begun, the general concensus of opinion seems to be that the physician should not divulge the nature of the case for prosecution, since the only wrong attached to this course consists in a failure to punish the guilty individual, already subjected to much suffering, or certain accomplices who cannot be punished without disgracing publicly, the unfortunate woman.

In regard to cases in which the physician is asked to aid in a positive deception, it seems to us that the ethical problem is somewhat different. It is one thing to preserve secrecy, another to assume an active role, in a fraud. The problem often assumes the form of upholding a superstitious chastity, tacitly or by explicit misstatement of facts, for the sake of securing marriage or preventing its disruption. This problem manifests itself in various forms, with circumstances sometimes prompting to sympathy with one party, sometimes with the other. A male physician naturally likes to stand by his own sex, a female by her own. Each may or may not be personally influenced by the time-honored social distinction of standards of sexual morality. Personal friendship or even close relationship may complicate the problem by prejudicing the physician in one direction or another. A further complication is added if the physician owes professional loyalty both to the patient and to the other party, and this complication often takes the particular form of being summoned by one party—say a husband or fiance—to attend a patient who, in turn, demands his co-operation in a deceit of the very one who is employing him and paying for his services. Now it seems to us that, however human the tendency to be swerved by personal and professional affiliations, there should be one rule to cover all cases of this general nature and that, it should neither require the violation of a professional confidence or observation nor involve the physician as an accessory to a deceit and fraud. It is comparatively easy to state the solution in general terms, but by no means so to lay down practical rules of ethical guidance. The only practical solution that occurs to us is a general policy to refuse a case, even in a purely advisory capacity, that will involve the attendant in duplicity, perhaps by a notice displayed in the office to all patients, or ready for display on first learning the general circumstances, in some such words as these: "Dr. ——————, appreciates the necessity of guarding any secrets disclosed by statements of patients or by their examination, or accidentally revealed during the course of his attendance, and will not reveal any such secret under ordinary circumstances, but refuses to aid, by any misstatement or concealment of facts, in the violation of law or in any deceit or fraud upon another person."

Our attention has been called to this general topic by the following three cases of overt professional assistance in deception: 1. Preparation of a capsule containing a red liquid, for use in vagina, to simulate haemorrhage from a ruptured hymen; 2. Plastic operation to restore ruptured hymen, successful, according to statement of patient, to the degree of resisting attempts to penetrate for three hours; 3. Man refusing to live with wife unless she would bear children. Previous total hysterectomy concealed from husband. Physician secured illegitimate child during husband's absence and assisted in palming it off on the husband as his own, by his wife. Elaborate details as to correspondence as to progress of pregnancy, subterfuges to keep husband away till after the fictitious puerperium, etc., reported in medical journal.

